# “Não sou antivacina, mas…”: entendendo a hesitação vacinal a partir das narrativas de pais hesitantes

**DOI:** 10.1590/0102-311XPT154624

**Published:** 2025-06-09

**Authors:** Kelly Simone Almeida Cunegundes, Daisy Maria Machado, Nadia Vitorino Vieira

**Affiliations:** 1 Universidade Federal de São Paulo, São Paulo, Brasil.

**Keywords:** Hesitação Vacinal, Movimento Antivacina, Cobertura Vacinal, Vaccination Hesitance, Anti-vaccine Movement, Vaccination Coverage, Vacilación a la Vacunación, Movimiento Anti-vacunación, Cobertura de Vacunación

## Abstract

A hesitação vacinal é um fenômeno relacionado ao receio, atraso, recusa parcial ou total das vacinas recomendadas pelos programas de imunização. Este fenômeno tem causado preocupação, pois ameaça conquistas importantes na redução da morbimortalidade das doenças imunopreviníveis. Os objetivos foram compreender o processo de decisão dos pais hesitantes sobre (não) vacinar e avaliar os significados que estes pais atribuem à vacinação, e quais vivências as decisões acerca da vacinação podem suscitar. Realizou-se abordagem qualitativa através de entrevistas individuais em profundidade realizadas entre setembro de 2020 e julho de 2021. A amostra foi constituída a partir da estratégia de bola de neve e para definir o tamanho da amostra foi usado o princípio da saturação teórica. Foram incluídos pais que se enquadravam na definição de hesitação vacinal, ou seja, atrasavam ou recusavam uma ou mais vacinas. As entrevistas foram gravadas digitalmente, transcritas *verbatim* e textualizadas. Para a análise das entrevistas foi empregada a fenomenologia interpretativa. Foram incluídos dez participantes, quatro temas emergiram das narrativas: estilo de vida natural; pressão social no contexto da vacinação (profissionais de saúde, familiares, amigos etc.); pandemia e vacinação contra COVID-19; e desconfiança nas vacinas, indústria farmacêutica, profissionais de saúde e instituições. A hesitação vacinal é um fenômeno complexo e multifacetado. É fundamental capacitar profissionais de saúde para dialogar com pais hesitantes no contexto da vacinação. A hesitação vacinal é um fenômeno volátil e contextos epidemiológicos diversos, como a pandemia, podem influenciar a reflexão de pais hesitantes sobre a vacinação de rotina.

## Abstract (en)

Vaccine hesitancy constitutes a phenomenon related to fear, delay, and partial or total refusal of vaccines recommended by immunization programs. This phenomenon has caused concern as it threatens important achievements regarding the reduction of the morbidity and mortality of vaccine-preventable diseases. This study aimed to understand the decision process of parents who were hesitant about vaccines. This study aimed to evaluate the meanings these parents attribute to vaccination and what experiences can be elicited by decisions about vaccination. A qualitative research was carried out by in-depth individual interviews from September 2020 to July 2021. Snowball sampling was employed, using the principle of theoretical saturation to define sample size. Parents who met the definition of vaccine hesitancy - who delayed application or refused one or more vaccines - were included in this study. Interviews were digitally recorded, transcribed verbatim, and turned into texts. Interpretative phenomenology was used to analyze the interviews with the 10 participants. Moreover, four themes emerged from their narratives: natural lifestyle; social pressure regarding vaccination (healthcare providers, family members, friends, etc.); the pandemic and vaccination against COVID-19; and distrust in vaccines, the pharmaceutical industry, and healthcare providers and institutions. Vaccine hesitancy constitutes a complex and multifaceted phenomenon. Healthcare providers must be trained to dialogue with hesitant parents regarding vaccination. This volatile phenomenon and diverse epidemiological contexts, such as the pandemic, can influence the reflection of hesitant parents about routine vaccination.

## Resumen

La vacilación vacunal es un fenómeno relacionado con el miedo, el retraso, el rechazo parcial o total de las vacunas recomendadas por los programas de inmunización. Este fenómeno ha causado preocupación porque amenaza importantes logros en la reducción de la morbimortalidad de las enfermedades prevenibles por vacunación. El objetivo fue comprender el proceso de decisión de los padres indecisos sobre (no) vacunar. Se buscó evaluar los significados que los padres atribuyen a la vacunación y qué experiencias pueden provocar las decisiones sobre la vacunación. Se realizó un estudio cualitativo mediante la aplicación de entrevistas individuales en profundidad entre septiembre de 2020 y julio de 2021. La muestra se constituyó a partir de la técnica bola de nieve; y para definir el tamaño de la muestra se utilizó el principio de saturación teórica. Se incluyeron a padres que cumplían con la definición de vacilación vacunal, es decir, retrasaron o rechazaron una o más vacunas. Las entrevistas fueron grabadas digitalmente para su posterior transcripción. Se utilizó fenomenología interpretativa para analizar las entrevistas. Se incluyeron diez participantes; y cuatro temas surgieron de los relatos: estilo de vida natural; presión social en el contexto de la vacunación (profesionales de la salud, familiares, amigos, etc.); pandemia y vacunación contra la COVID-19; y desconfianza en las vacunas, la industria farmacéutica, los profesionales de la salud y las instituciones. La vacilación vacunal es un fenómeno complejo y multifacético. Es esencial que los profesionales de la salud estén capacitados para conversar con los padres indecisos en el contexto de la vacunación. La vacilación vacunal es un fenómeno volátil y diversos contextos epidemiológicos, como la pandemia, pueden influir en la reflexión de los padres indecisos sobre la vacunación de rutina.

A vacinação é considerada uma das maiores conquistas da medicina moderna. Os programas de imunização em massa tiveram considerável êxito na erradicação, controle e eliminação de doenças infectocontagiosas ao longo de décadas ^1^ ^,^ ^2^. Apesar dessa história de sucesso, nos últimos anos, muitos pais têm questionado a importância da vacinação, resultando nas quedas das coberturas vacinais e o reaparecimento de doenças infectocontagiosas que já haviam sido controladas ^1^ ^,^ ^2^ ^,^ ^3^ ^,^ ^4^ ^,^ ^5^ ^,^ ^6^.

Em 2014, a Organização Mundial da Saúde (OMS) definiu que o atraso ou a recusa de vacinas, apesar da disponibilidade, é um fenômeno denominado hesitação vacinal ^2^. O termo abriga um amplo espectro de comportamentos e atitudes em relação à vacinação. É um fenômeno complexo com causas multidimensionais e que variam conforme a época, vacina, local e contexto ^2^ ^,^ ^3^. Cinco fatores estariam implicados na hesitação vacinal: confiança, complacência (não acreditam na necessidade da vacinação), conveniência (acesso, custos, barreiras estruturais e psicológicas), responsabilidade coletiva e cálculo (busca de informações, ponderação de riscos e benefícios) ^2^ ^,^ ^3^ ^,^ ^4^. 

A pandemia de COVID-19 mostrou o quão volátil a discussão sobre vacinas pode ocorrer na arena pública ^7^. A revolução digital impõe enormes desafios à comunicação em saúde pública, pois a exposição às redes sociais favorece, desproporcionalmente, a hesitação vacinal em contraposição à aceitação vacinal, visto que os conteúdos antivacina engajam muito mais do que os conteúdos pró-vacinas ^8^ ^,^ ^9^ ^,^ ^10^ ^,^ ^11^. 

O Fundo das Nações Unidas para a Infância (UNICEF) chama atenção, em relatório de 2023, não apenas para as quedas das coberturas vacinais, mas também para indicadores que mensuram a confiança nas vacinas em diversos países e regiões. Dos 55 países analisados, em apenas três foi observado aumento na confiança nas vacinas, todos os demais países apresentaram queda desse indicador ^12^. 

No Brasil, ao longo de décadas, foi-se estabelecendo uma forte cultura de imunização. As bem-sucedidas campanhas de vacinação contra varíola e poliomielite, a partir dos anos 1960, contribuíram para a normalização da adesão à vacinação na sociedade brasileira. O Programa Nacional de Imunizações (PNI), criado em 1973, conseguiu atingir resultados sustentáveis por décadas. O estabelecimento do Sistema Único de Saúde (SUS) garantiu o progressivo aumento da quantidade de imunizantes e da cobertura vacinal através da vacinação de rotina e das campanhas ^13^. 

Contudo, a partir de 2016, observou-se que as coberturas vacinais não atingiram as metas preconizadas. Em 2017 constatou-se a queda entre 18 e 21 pontos percentuais das coberturas vacinais para seis vacinas (poliomielite, hepatite A, meningocócica C, pentavalente, rotavírus e hepatite B) ^14^. As razões para esta tendência podem se relacionar a vários fatores, tais como enfraquecimento do SUS, a implantação de sistema de informações do PNI, aspectos sociais e culturais. Entretanto, não parece ser desprezível a influência da hesitação vacinal. Além disto, a Organização Panamericana da Saúde (OPAS) aponta que, no Brasil, houve uma redução de 10% da confiança nas vacinas ^12^ ^,^ ^13^ ^,^ ^14^. 

Em inquérito de cobertura vacinal, realizado em todas as capitais do Brasil, a análise de uma coorte de nascidos entre 2017 e 2018, demonstrou que as coberturas para o esquema de vacinação era adequado em apenas 75% da amostra. Além disto, em 17 capitais da federação, as coberturas eram menores em estratos socioeconômicos mais altos quando comparados com os estratos mais baixos ^15^. A mesma tendência foi observada em inquérito de cobertura vacinal na cidade de São Paulo ^16^.

Entender o processo de decisão de vacinar ou não sob a perspectiva dos pais é crucial. Investigar os valores, medos, (des)informações e vivências que moldam a decisão de não vacinar de diversos subgrupos pode ajudar a formular estratégias educacionais e dialógicas que permitam pensar e por em prática soluções efetivas ^17^ ^,^ ^18^.

Os objetivos foram investigar e refletir sobre o processo de tomada de decisão de (não) vacinar os filhos de pais cujo comportamento em relação à vacinação se enquadram na definição de hesitação vacinal. Compreender quais fatores influenciam as decisões dos pais hesitantes, quais significados estes pais atribuem à vacinação e quais vivências estas decisões ocasionam. 

## Percurso metodológico

Neste estudo foi utilizada a metodologia qualitativa. Foram realizadas entrevistas individuais em profundidade entre setembro de 2020 e julho de 2021 (durante a pandemia de COVID-19). Foram considerados elegíveis pais ou tutores, maiores de 18 anos, que atrasavam ou recusavam a administração de uma, algumas ou todas as vacinas. Os pontos de partida no processo de inclusão foram dois grupos de WhatsApp. Um dos grupos era composto de pais de uma escola privada com pedagogia alternativa, pedagodia Waldorf, na cidade de São Paulo, pois neste tipo de escola observa-se significativa proporção de pais que apresentam hesitação vacinal ^17^ ^,^ ^18^ ^,^ ^19^ ^,^ ^20^; e o outro grupo de WhatsApp de infectopediatras. A identidade de todos os participantes foi protegida através da adoção de pseudônimos. 

A amostragem foi constituída pela abordagem de bola de neve que é uma forma de amostra não-probabilística que mostra-se útil para estudar grupos difíceis de acessar e que o pesquisador pode se beneficiar do ciclo social dos entrevistados ^21^. O tamanho da amostra foi definido pelo critério da saturação teórica ^22^ ^,^ ^23^. As entrevistas foram gravadas digitalmente e transcritas *verbatim*. 

Foi empregada a fenomenologia interpretativa a fim de definir os temas que emergiram das entrevistas ^24^. A pesquisa foi aprovada pelo Comitê de Ética em Pesquisa da Universidade Federal de São Paulo - Unifesp (Projeto CEP/Unifesp nº 0154/2020, Parecer nº 4.055.173). Todos os participantes assinaram o Termo de Consentimento Livre e Esclarecido.

A investigadora principal (K.S.A.C.) que conduziu as inclusões dos participantes fazia parte da comunidade escolar Waldorf na cidade de São Paulo, pois tinha uma filha estudando naquele estabelecimento. Além disto, a mesma pesquisadora era infectologista pediátrica. Estar inserida nesta comunidade facilitou o acesso aos participantes do estudo, por outro lado, impõe ao pesquisador uma postura de reflexão crítica permanente sobre o seu papel como pesquisador tentando “olhar de fora” ao mesmo tempo estando “dentro” ^25^.

## Resultados

Foram incluídos dez participantes. As entrevistas tiveram duração média de 1 hora e 20 minutos. No Quadro 1 estão as características dos participantes do estudo. Dois dos entrevistados são genitores e médicos (homeopata e o outro pediatra antroposófico). Todos os participantes tinham pelo menos o nível superior e dois participantes tinham doutorado. 

**Quadro 1:** Características dos participantes incluídos no estudo.

NÚMERO	CODINOME	ENTREVISTA	IDADE	GÊNERO	PROFISSÃO	FILHOS	IDADES (ANOS)	RESIDÊNCIA
1	Julia	Presencial	40	Feminino	Psicóloga	2	7, 11	São Paulo
2	Henrique	Online	39	Masculino	Consultor fiscal	1	4	São Paulo
3	Danilo	Presencial	37	Masculino	Gerente de TI	1	3, 1	São Paulo
4	Saulo	Presencial	58	Masculino	Médico	3	20, 18, 13	São Paulo
5	Laura	Online	42	Feminino	Psicóloga	1	4	Florianópolis
6	Clarice	Online	43	Feminino	Professora	2	5, 8	São Paulo
7	Patricia	Online	44	Feminino	Jornalista, Cientista social	2	9, 11	Holambra (São Paulo)
8	Paula	Online	47	Feminino	Médica	1	14	Jundiaí (São Paulo)
9	Roberto	Presencial	39	Masculino	Marketing	3	9, 5, 2	Holambra (São Paulo)
10	Brahma	Online	45	Masculino	Professor de yoga	4	13, 11, 3, 1	Monteiro Lobato (São Paulo)

Todos as crianças cujos pais participaram do estudo e estavam em idade escolar estavam matriculados em escolas privadas. Sete participantes tinham filhos frequentando escolas da pedagogia Waldorf.

Após análise das narrativas foram identificados quatro temas: estilo de vida natural; pressão social; pandemia de coronavírus e vacinação contra coronavírus; e desconfiança nas vacinas, nos profissionais e instituições.

### Estilo de vida natural

Observou-se uma forte associação entre a adoção de um estilo de vida natural com sentimentos e comportamentos relacionados à hesitação vacinal. Segundo os pais, a adoção de certos hábitos na vida cotidiana poderia garantir bem-estar e equilíbrio e, portanto, prevenir doenças, sendo muitas vezes, considerado que alguns hábitos poderiam tornar medidas preventivas desnecessárias. São elencados como hábitos saudáveis: alimentos orgânicos; contato com a natureza; meditação; medicamentos alternativos, como homeopáticos, antroposóficos, fitoterapia etc. Muitas vezes o uso de medicações alopáticas ou outras intervenções da medicina tradicional são vistas como “contaminantes” desse ideal de vida natural. Conforme afirmação de um dos participantes: “*A vacina me soa uma coisa antinatural, mas as pessoas estão vivendo de uma forma antinatural também*” (Brahma, professor de yoga).

As vacinas e seus efeitos são vistos como uma intervenção num processo natural, conforme revela um dos participantes.

“*O corpo tem que passar por um problema e aprender com este problema, imunologicamente falando, no desenvolvimento geral. Todo aprendizado tem crise, tem problema, tem suas consequências… as crises, na expressão física, seriam as doenças, então as doenças também podem trazer coisas boas, não só ruins* (Saulo, pediatra antroposófico). 

A adoção de abordagens médicas complementares permite aos participantes conferir significados diversos às doenças infectocontagiosas. A vacinação, algumas vezes, é vista pelos participantes como desnecessária ou uma intervenção que produziria uma resposta (artificial) inferior à proteção produzida pela doença (natural). Outra questão presente nas narrativas é que as doenças infectocontagiosas são consideradas normais ou “próprias da infância” e, portanto, também “naturais” no decorrer da vida. A percepção de que a doença imunoprevinível é um evento positivo sublinha o simbolismo do que é ter uma vida natural, que vai além da adoção de práticas saudáveis para evitar algumas doenças, mas inclui também ficar doente na infância para garantir uma imunidade “verdadeira”. Conforme afirma o participante: “...*Teve um surto de sarampo e a maioria das crianças do prédio foram vacinadas, os nossos filhos não foram vacinados.* (...) *Qual o problema de eles pegarem sarampo? É uma imunidade natural, uma imunidade verdadeira*” (Danilo, gerente de TI).

A percepção dos pais do perigo de contrair as doenças infecciosas difere daquilo que a medicina alopática considera risco, pois este último está ancorado em estatísticas, enquanto que na percepção dos pais hesitantes, muitas vezes, o risco benefício estatístico é secundário, ou mesmo, nem aparece em seus horizontes de decisão.

### Pressão social

É muitas vezes durante o pré-natal que os pais começam a pensar na vacinação dos seus filhos. No contexto do planejamento de um parto natural os pais se inserem numa cultura de questionamentos das intervenções da medicina alopática. A complexidade do calendário de vacinação da infância impõe a estes pais uma busca por profissionais de confiança que possam dialogar e ajudá-los a decidir o melhor caminho. Para outros, a opinião de um profissional de confiança é um dos aspectos da busca por informação. 

A busca por um parto natural muitas vezes introduz os pais numa abordagem médica alternativa que apoia decisões diferentes dos protocolos adotados como, por exemplo, a recusa da vacinação contra hepatite B e BCG no berçário. A individualização não se restringe à vacinação, mas também a outras condutas praticadas em maternidades, como administração de vitamina K (prevenção da doença hemorrágica do recém-nascido) e aplicação do colírio de nitrato de prata (prevenção da conjuntivite por gonococo). 

Os pais relatam que muitas vezes estas escolhas individualizadas colidem com protocolos institucionais e causam discussões e confrontos com os profissionais de saúde. Alguns pais reclamam da falta de diálogo e da falta de preparo dos profissionais para lidar com situações em que a conduta predominante é questionada. Outros relatam uma postura confrontacional e policialesca. Conforme o participante a seguir descreve a sua experiência em maternidade: “...*Depois que* [o filho] *nasceu a gente ainda teve todo um cuidado porque não queríamos que pingasse o colírio, o nitrato de prata.* (...) *Então várias coisas que a gente falou: ‘Não, a gente não quer que aconteça isso’. ‘Então assina esse termo aí’. ‘Tá bom, eu assino’. Mas fica um processo tão ruim que a gente depois tratou tudo em uma ouvidoria do hospital.* (...) *A experiência não foi boa, foi meio tenso, a gente não gosta de lembrar*” (Roberto, marketing).

Recorrer a um serviço de saúde (público ou privado) para vacinar quando algumas vacinas não estão em dia ou há recusa de uma ou mais vacinas pode também ser um momento de interação com o profissional de saúde que mais se aproxima de um embate. A participante Júlia relata sua experiência em clínica de vacinação privada: “*Quando surgiu a questão da febre amarela, eu fui a uma clínica de vacinação privada para dar a vacina de febre amarela.* (...) *O médico que nos atendeu falou - ‘você esqueceu a carteirinha dela em casa? Por que… o quê que é isso?!’. E eu querendo resolver a questão da febre amarela, mas o médico que me atendeu foi super irônico e na frente das minhas filhas*” (Julia, psicóloga).

O participante Roberto relata que quando vai à unidade básica de saúde (UBS) dar vacinas que estão atrasadas, a profissional de saúde da unidade fala - está tão atrasada que eu não sei se eu posso dar. O mesmo participante relata que as idas à UBS para vacinar eram marcadas por muita tensão. Ao ser questionado pela profissional de saúde sobre os atrasos na vacinação, o participante rebateu: “*A vacina não é uma obrigação por enquanto; ela é um direito, mas não é obrigatória*”*.* E ela falava: “*Mas vocês estão fazendo tudo errado. Isso aqui está contra o Ministério, não pode*”. 

Nesse caso, o participante considera apenas o direito à proteção individual e desconsidera a obrigatoriedade da vacinação vigente na legislação brasileira que visa garantir a contribuição individual na imunidade coletiva ^26^. Já para a participante Paula, o reconhecimento da obrigatoriedade da vacinação é motivo de grande preocupação “*Eu tenho um medo muito grande, muito grande mesmo em relação ao conselho tutelar. Então, ficar na clandestinidade é uma coisa que me preocupava muito em relação a ela* [a filha]” (Paula, médica).

O participante Saulo, médico antroposófico, relata que as vivências de suas pacientes que buscam serviços de saúde com a vacinação atrasada é marcada pelo julgamentos e ameaças. É comum entre os seus pacientes que atrasam a vacinação serem ameaçados na unidade básica de serem denunciados ao conselho tutelar. Em algumas ocasiões essas ameaças se concretizam e os pais hesitantes são abordados por conselheiros tutelares. 

Num contexto social, com amigos e familiares, alguns pais procuram evitar o tema vacinação para não gerar discussões polêmicas; quando perguntados sobre o assunto, preferem mentir. Uma das participantes, Paula, relatou que o questionamento sobre vacinação partiu da própria filha de dez anos, que, após fazer um trabalho na escola sobre vacinas, questionou a conduta dos pais a partir do conteúdo discutido em sala de aula, alegando que havia aprendido que a vacinação era importante e que os pais haviam tomado a decisão errada ao não vaciná-la.

Observa-se discordâncias até mesmo entre pessoas do mesmo grupo que questionam a decisão de outras mães de não vacinar. Há também o reconhecimento entre pais de que escolhas sobre (não) vacinação estão associadas ao privilégio econômico. 

### Pandemia e vacinação de COVID-19

Em vista do contexto em que as entrevistas foram realizadas, entre 2020 e 2021, em meio a um intenso debate público sobre vacinas contra COVID-19, todos os participantes levantaram questões sobre a pandemia e vacinação contra COVID-19. Muitos pais relataram interesse em receber a vacina contra COVID-19, embora alguns tenham manifestado cautela alegando pouco tempo de desenvolvimento da mesma. A polarização política na pandemia levou alguns participantes a refletir sobre o pacto social da vacinação, e faziam questão de declarar que não se viam identificados com o posicionamento político daqueles que propagavam informações falsas sobre vacina contra COVID-19.

Para Paula, o intenso debate político-ideológico no contexto da pandemia a fez repensar sobre sua posição acerca da vacinação de rotina da própria filha. 

“*Um argumento que eu conversei com os meus colegas do grupo de homeopatia foi que ir contra a vacina nos alinhava, nos colocava no mesmo saco, com grupos políticos negacionistas. Eu não acho que o meu pensamento se alinha ao deles!*” (Paula, médica).

Os participantes frequentemente relataram que a pandemia de COVID-19 suscitou reflexões sobre o aspecto relacional das doenças infectocontagiosas. A discussão sobre grupos de risco para COVID-19 favoreceu o entendimento e revigorou a discussão sobre imunidade coletiva, reforçando a ideia de que a decisão de (não) vacinar não se situa apenas na esfera individual. 

“...*Então, é claro que nessa pandemia eu estou rezando para que essa vacina chegue logo, e eu vou tomar, meu marido vai tomar e todo mundo vai tomar, e me fez pensar bastante, por exemplo, na vacina de catapora, o quanto talvez tenha sido um egoísmo meu não dar para os meus filhos, pensando que uma criança pobre pode pegar e morrer. Acho que trouxe uma consciência muito grande essa pandemia*” (Clarice, professora).

“...*se não tomar* [vacina] *de COVID-19, eu posso transmitir para alguém. Eu acho que isso para mim é uma variável importante na hora da decisão. Eu entendo a vacina como um pacto da sociedade para eliminar uma doença, não quero ser uma transmissora da doença*” (Laura, psicóloga).

### Desconfiança nas vacinas, nos profissionais e instituições

Os participantes manifestam diferentes graus de desconfiança para não aderirem à vacinação. Muitos questionam o quanto interesses econômicos podem influenciar a indicação de vacinas por instituições de saúde pública. A indústria farmacêutica é vista como um segmento poderoso e totalmente dirigido por interesses econômicos e muitos questionam a autonomia dos profissionais de saúde de questionar este sistema. Esta percepção fica evidente nesta fala de um dos participantes: “...*os dois maiores setores financeiros do mundo hoje é o setor bélico e o farmacêutico. As pessoas não têm interesses financeiros!?*” (Danilo, gerente de TI). Outra participante sublinha a sua desconfiança na indústria farmacêutica: “...*apesar de eu acreditar na ciência e ser uma pessoa que acha que a ciência tem que ser o canal principal para a gente seguir algumas coisas, eu não confio na indústria farmacêutica e nem na classe médica*” (Laura, psicóloga).

Apesar da associação entre autismo e vacinas ter sido exaustivamente desacreditada, esta foi uma questão levantada por dois participantes. Geralmente esta associação relaciona-se com fontes de grupos antivacina e notícias falsas. Curiosamente, esses relatos partiram de pais que diziam buscar informações independentes e imparciais através de internet e livros. Entretanto, em suas narrativas, encontrava-se um farto repertório de desinformação e *fake news*. 

Uma participante, que era aderente à vacinação, descreveu que sua trajetória de hesitação vacinal começou após a filha, aos dois meses de idade, ter tido uma reação importante à primeira dose da vacina DTP (difteria, tétano e coqueluche), evento que ocasionou atendimento em um serviço de emergência. Ela relata que a falta de diálogo sobre eventos adversos e a falta de acolhimento a deixou traumatizada e perdeu a confiança na vacinação e no suporte que os profissionais de saúde poderiam dar diante de um novo evento adverso.

## Discussão

Neste estudo, procurou-se entender com maior profundidade a hesitação vacinal a partir da perspectiva de pais altamente escolarizados. O fenômeno de hesitação vacinal alberga um mosaico de atitudes e sentimentos em relação à vacinação e conhecer as particularidades de diversos grupos pode ajudar a construir diálogos e estratégias mais efetivas ^4^.

Todos os participantes recusaram o rótulo “antivacina”. Muitas são as alegações que se seguem à afirmação “não sou antivacina...”, por exemplo, a oposição à obrigatoriedade da vacinação, a padronização do esquema de vacinação, o número de vacinas recomendadas e as idades recomendadas para a vacinação que estes participantes consideram muito cedo, a aplicação de muitas vacinas simultaneamente, a concepção de que a vacinação não é necessária pois o sistema imune já seria suficientemente competente. Outro fator a se considerar é que, com a polarização político-ideológica durante a pandemia de COVID-19, muitos pais hesitantes pareciam fazer questão de deixar claro que seus comportamentos em relação à vacinação não se alinhavam com o discurso antivacinação que era ventilado intensamente naquele contexto ^27^. 

Nesse estudo, quatro temas emergiram das narrativas: estilo de vida natural; pressão social; pandemia vacinação de COVID-19; e desconfiança nas vacinas, em profissionais de saúde e indústria. A escolha de (não) vacinar parece fazer parte de um conjunto de outras escolhas que constituem uma identidade social mais ampla. Estudos apontam para uma forte associação de pais de classe média com a adoção de hábitos tidos como naturais e/ou saudáveis ^18^ ^,^ ^19^ ^,^ ^20^. O termo “parentalidade salutogênica” surgiu para definir pais empenhados na adoção de hábitos saudáveis que, segundo eles, poderiam garantir um sistema imune mais apto para enfrentar as doenças. Sendo assim, a busca por parto natural, amamentação prolongada, alimentação orgânica, educação alternativa e medicina complementar fazem parte desse modelo de parentalidade. É marcado também por intensa crítica e questionamento do modelo biomédico ocidental e suas intervenções ^28^. 

Estudo sobre hesitação vacinal ^29^ demonstrou nas narrativas dos pais uma dicotomia entre o natural e o artificial e a vacinação é vista como desnecessária e indesejável, visto que as infecções, sendo naturais, promoveriam uma proteção superior sem que houvesse necessidade de exposição às vacinas que, para esses pais, são produtos artificiais/tóxicos. Outro estudo ^30^ evidenciou que as decisões relacionadas à saúde dos filhos associava-se à resistência e crítica ao paradigma médico normativo que acusavam de negligenciar a totalidade e a singularidade do indivíduo. 

Uma das características de alguns participantes nesta pesquisa é a adesão a modelos pedagógicos alternativos. Dos dez participantes, sete têm filhos em escolas da pedagogia Waldorf, cujas raízes remontam à Alemanha do início do século passado, quando o austríaco Rudolf Steiner (1861-1925) estabeleceu a antroposofia. A antroposofia se baseia na crença de que o universo espiritual e material se interrelacionam ^19^. A pedagogia Waldorf é um método que valoriza o papel das artes no aprendizado, reconhece o processo experencial na formação e se caracteriza por um ensino não intervencionista que valoriza a capacidade individual. Esses ambientes escolares se alinham a estas características na medida em que o “natural” prevalece, como brinquedos de madeira, alimentos orgânicos e ausência de eletrônicos ^20^. Segundo Sobo ^31^, a discussão sobre vacinação em escolas Waldorf é profundamente influenciada pelo círculo social. 

Neste estudo, verificou-se uma grande afinidade dos pais com práticas de saúde complementares. É marcante o relato dos pais hesitantes de encontrar nos profissionais de saúde que adotam as terapias complementares ressonância com as suas escolhas em relação à vacinação. Alguns pais não fazem uma busca ativa de informações sobre vacinas, mas apenas confiam nas recomendações de médicos que os auxiliam na escolha de quais vacinas e o momento em que estas devem ser dadas. Outros pais fazem a própria busca de informações e consideram também a opinião de profissionais. 

Os pais relatam que os atendimentos em serviços de saúde públicos ou privados que adotam a medicina alopática, no contexto da vacinação, são geralmente experiências marcadas por julgamentos, críticas e até ameaças. Há muitos relatos na literatura sobre a estigmatização dos pais hesitantes por profissionais de saúde. É reconhecido ainda que experiências negativas nessas interações podem afastar os pais de serviços de saúde e causar enorme frustração, tanto para os pais quanto para os profissionais de saúde, que se sentem incomodados com questionamentos e eventualmente com a recusa dos pais em seguir as orientações ^18^ ^,^ ^19^ ^,^ ^20^ ^,^ ^30^ ^,^ ^31^ ^,^ ^32^ ^,^ ^33^.

Em pesquisa realizada na Austrália, observou-se que a qualidade das interações em serviços de saúde pode influenciar as decisões sobre vacinação. Constatou-se ainda que decisões sobre não vacinar muitas vezes derivam de experiências negativas em serviços de saúde relacionados a outros contextos de assistência que afastam os pais de serviços de saúde e suas intervenções, inclusive vacinas ^34^. A mitigação da ansiedade em relação à vacinação requer que o profissional de saúde tenha uma atitude empática, escuta atenta, respeitosa e clareza quanto aos eventos adversos e ao suporte que pode ser oferecido caso estes ocorram ^35^ ^,^ ^36^ ^,^ ^37^. 

Em revisão sistemática, discute-se duas possíveis vertentes para o entendimento da hesitação vacinal. O primeiro conceito é que a lógica neoliberal sugere que as decisões em saúde sejam entendidas no âmbito do risco, escolha e responsabilidades individuais. Essa lógica entra em conflito com os programas de imunização em massa na medida em que os últimos enfatizam a saúde coletiva. O segundo conceito é de exclusão social. Em países de média e baixa rendas, as experiências de exclusão social podem comprometer a adesão a programas de imunização e inocular desconfiança em governos e programas públicos ^38^. 

A pandemia de COVID-19 suscitou reflexões acerca do pacto coletivo na vacinação. Este achado é interessante, pois o papel da imunidade coletiva mostra-se um motivador opaco dentre as razões que levam os pais a vacinar, mesmo aqueles aderentes o fazem primordialmente pela proteção individual ^39^. Em revisão sistemática, observou-se que 1% a 6% dos pais considerava a proteção coletiva como fator principal para vacinar seus filhos enquanto que 37% considerava fator secundário ^40^. 

Um dos participantes apresentou em sua narrativa conteúdos religiosos mais conservadores. Em estudo realizado no Brasil, que avalia a hesitação vacinal para a vacina de COVID-19, observou-se uma forte associação de ultraconservadorismo e religião evangélica com a desconfiança e à recusa desta vacina ^41^. A intersecção entre hesitação vacinal e religião tem sido observada, sobretudo durante a pandemia, mostrando a necessidade de ajustar as campanhas e diálogos com grupos religiosos específicos considerando suas crenças e buscar interlocução com líderes de comunidades religiosas ^42^. 

Na trajetória de decisão sobre vacinação, os pais reavaliam suas escolhas sobre vacinas mediante diversos fatores, como experiências com eventos adversos em pessoas próximas ou em seus próprios filhos, contextos epidemiológicos como surtos ou epidemias que revigoram a importância de estar protegido contra determinada doença imunoprevinível e questões relacionadas à comunicação com profissionais de saúde no contexto da vacinação ^43^. 

Algumas atitudes de profissionais de saúde com pais hesitantes influenciaram positivamente a decisão dos pais acerca da vacinação, por exemplo: escuta respeitosa dos receios e dúvidas sobre vacinação, comunicar valores compartilhados, compartilhar experiências pessoais com vacinação e doenças imunopreviníveis, explicar a racionalidade envolvida na elaboração dos calendários de vacinação e abordar medos específicos em relação aos ingredientes das vacinas e eventos adversos. Por outro lado, observa-se que quando as dúvidas e anseios não são adequadamente abordadas durante o pré-natal ou na ocasião do nascimento da criança, mesmo que estas mães inicialmente aceitem a vacinação, há tendência para que esta jornada posteriormente entre na rota da hesitação vacinal ^34^ ^,^ ^35^ ^,^ ^37^ ^,^ ^41^.

No Brasil, estudo sobre hesitação vacinal ^44^ constatou que pais começam a buscar informações a partir da inserção no contexto do parto humanizado e utilizam a internet como fonte de informação sobre vacinas. Além disso, muitas vezes pais hesitantes omitem a situação vacinal de seus filhos em seus ciclos sociais com medo de cobranças, polêmicas e exclusão ^45^. Para muitos pais hesitantes, não vacinar é uma decisão vista como uma manifestação do cuidado parental, visto que estes pais se sentem “protegendo” os filhos dos supostos malefícios das vacinas ^46^ ^,^ ^47^ ^,^ ^48^. 

Este estudo permitiu aprofundar o significados e vivências de pais hesitantes à vacinação. Traz informações relevantes sobre aspectos que influenciam a decisão dos participantes e, por ter sido realizado durante a pandemia, propiciou uma nova camada de análise do fenômeno, pois favoreceu novas reflexões desses pais sobre a dimensão coletiva da imunização. Algumas limitações devem ser consideradas, como o fato de investigar o fenômeno num recorte social específico, pois todos os participantes têm filhos em escolas privadas, são de nível socioeconômico mais elevado; e a maioria é procedente de escolas Waldorf, não sendo possível extrapolar estes achados para outros contextos socioculturais. 

Muito se discute qual seria a melhor estratégia de comunicação para abordar as desinformações, dúvidas, rumores e mentiras acerca das vacinas. Levando em consideração a heterogeneidade do fenômeno, assume-se que uma estratégia eficaz num subgrupo não teria resultados positivos em outro subgrupo. As mensagens que exaltam o medo das doenças infectocontagiosas parecem ter um efeito oposto em grupos mais aguerridos à ideia de que a vacinação é um malefício, podendo inclusive aumentar a desconfiança ^49^. Estratégias promissoras incluem a comunicação clara e assertiva de consensos científicos e ágil reação às *fake news* difundidas em ambientes digitais ^8^ ^,^ ^9^ ^,^ ^10^. No recorte analisado neste estudo, a experiência da pandemia de COVID-19 suscitou em alguns participantes uma reflexão sobre a responsabilidade coletiva da vacinação. No entanto, não é possível afirmar que o mesmo ocorreria em outros grupos. Cumpre salientar que é inegável que a dimensão coletiva da vacinação deve ser fortemente considerada em políticas públicas de conscientização da população sobre vacinação. Além disto, reiterar que a vacinação um direito, mas, acima de tudo, um pacto social. 

Iniciativas como a do Município de São Paulo, com a Declaração de Vacinação Atualizada (DVA), documento que deve ser emitido pelas salas de vacina e permitirá a monitorização vacinal de estudantes do Ensino Fundamental, merecem destaque, mas abrange somente os alunos da rede municipal de ensino, e não alcança alunos da rede privada ^50^. Embora medidas como esta sejam importantes, não se deve perder de vista aspectos da comunicação individual, visto que o encontro entre profissional de saúde e pais hesitantes no contexto da vacinação pode ser marcado por muitas tensões. Algumas estratégias são estudadas para basear o diálogo acerca da vacinação com pais hesitantes. Recomenda-se considerar cada diálogo em sua singularidade e não simplesmente assumir que uma única estratégia se aplica a todos, sendo fundamental reconhecer os subgrupos e suas identidades culturais nessa comunicação ^8^. 

## Considerações finais

A hesitação vacinal é um fenômeno complexo, que coloca em risco décadas de avanços no enfrentamento de doenças infectocontagiosas. Observou-se que a comunicação, num nível individual e coletivo, precisa ser aprimorada. Estamos diante de uma mudança de paradigma, em que os questionamentos dos pais acerca da vacinação desafiam a percepção histórica na sociedade brasileira da vacinação como um ato normalizado. Faz-se também necessário capacitar profissionais para entender este fenômeno e incentivar a construção de diálogos com pais hesitantes nos serviços de saúde. É fundamental rever a postura paternalista e policialesca do profissional de saúde e reconsiderar o treinamento de profissionais de saúde em vacinação para além do conhecimento sobre o calendário de vacinação.
